# The Applications of Bone Marrow-Derived Stem Cells to Induce Tolerance and Chimerism in Organ Transplantation

**Published:** 2010-11-01

**Authors:** M. Ebrahimi, N. Aghdami

**Affiliations:** Department of Regenerative Medicine, Royan Institute for Stem Cell Biology and Technology, Tehran, Iran

**Keywords:** Organ transplantation, Mixed chimerism, Mesenchymal stem cells

## Abstract

Progress in understanding the cellular and molecular biology of the immune system, in the second half of the 20^th^ century brings the transplantation of replacement organs and tissues in clinical reality to cure disease. Immunosuppressive agents that are part of nearly every transplantation procedure, are toxic to some extent and their chronic use predisposes the patient to the development of infection and cancer. Alternatives to immunosuppression include modulation of host immune system to reduce the immune response and the induction of a state of immunologic tolerance. Induction of hematopoietic mixed chimerism through donor bone marrow transplantation offers a promising approach for tolerance induction as a prelude to organ transplantation. Furthermore, mesenchymal stromal cells have important effects on the host immune system and possess immune modulation properties that make them attractive for potential use in organ transplantation as immunosuppressant. Both modalities might potentially provide novel therapeutic options for treatment/prevention of rejection and/or repair of organ allografts through their multifaceted properties. In this review, evidences for the tolerogenic properties and mechanisms of hematopoietic mixed chimerism as well as mesenchymal stromal cells effects on allograft surveillance are summarized.

## INTRODUCTION

Organ transplantation is the only definite treatment for improving both the quality of life and survival in patients diagnosed with various critical diseases of the liver, kidneys, heart and other organs [1,2]. Alloimmunity of the recipient is the main barrier against successful transplantation and drug-induced non-specific immunosuppressive strategies are the only way to overcome the immune rejection process. Immunosuppressive regimens employed for solid organ transplantation are generally classified as induction, maintenance, or rescue therapies. The goal of induction immunosuppression is preventing acute rejection (AR) and ultimately facilitating a tolerogenic state, so severe prophylactic therapy is administered at the time of transplantation. Although, induction therapy is potent, its prolonged use is exceedingly toxic. Maintenance therapy is used when the degree of alloimmunity is diminished. While it is less potent than the induction therapy, it is generally required for the remainder of the life of the allograft [3]. Finally, rescue therapy is similar to the induction therapy in its intensity, but it is used in the setting of an episode of acute humoral or cellular rejection. However, modern immunosuppression is very effective in preventing AR episodes; non-specific chronic immunosuppression is accompanied by severe morbidity and mortality and does not effectively prevent chronic graft loss. In addition, diabetes mellitus, hypertension, neurotoxicity, malignancies, infections, and severe nephrotoxicity are all associated with immunosupression [4,5]. Recently, morbidity and a high risk of chronic graft failure could be avoided with the induction of specific tolerance towards donor antigens. To potentially overcome this dilemma, cell-based approaches are being explored to improve not only outcomes but also safety profiles, at least with a marked reduction in long-term immunosuppression.

In transplantation tolerance, which is “the survival of foreign tissue in normal recipients in the absence of immunosuppression,” both donor tissue and the host immune system are two foci for induction of tolerance. Although specific protocols such as reduction of organ antigenicity or antigen masking are used as donor tissue related methods, most tolerance-induction methods require activation—or at least ongoing involvement—of the host immune system. 

It has been reported that passenger leukocytes from donor-tissue can migrate after organ transplantation and successfully induce hypo-responsiveness due to micro-chimerism which may be essential for sustained allograft survival and with graft survival [6]. However, the use of micro-chimerism to induce tolerance is controversial. Furthermore, it has been noted that presence of donor cells outside the graft affect the long-term outcome of the allografts [7].

It is believed that infused donor bone marrow cells (DBMCs) contain hematopoitic stem cells that produce immune and blood cells. Immune cells such as dendritic cells, induce tolerance. It also consists of mesenchymal stem cells (MSCs) with capability of immunomodulation in organ transplantation. In this review, we focus on recent data describing the basic biology, experimental outcomes, and clinical implications of tolerance induction using bone marrow cells (BMCs) in organ transplantation.

**CHIMERISM**

The Chimera of Greek mythology was said to possess the head of a lion, the body of a goat and the hindquarters of a dragon. In human, “chimerism” is referred to as “a state in which an organism possesses cells derived from two or more distinct zygote lineages.”

Human chimeras were first discovered with the advent of blood typing, when it was determined that some people had more than one blood type. However, a new definition of chimerism is seen when foreign (donor) hematopoietic cells are present in an individual. Based on the percentage of donor cells, the state of chimerism can be categorized as “complete chimerism” in which 100% of the recipient blood cells have originated from the donor or “mixed chimerism” which donor cells are present in >1% of the recipient’s total cell population [8]. 

The relationship of chimerism to tolerance was discovered when Owen and colleagues observed that fully major histocompatibility complex (MHC)-mismatched skin graft was accepted between particular sets of twin cattle. Based upon this observation, set of animal experiments was done and revealed that hematopoietic chimerism induced in immunologically immature fetal or neonatal animals, lead to transplantation tolerance [9,10]. Subsequently, it was shown that tolerance could be achieved by bone marrow transplantation (BMT) to adult rodents whose immune and hematopoietic systems were first ablated with lethal total body irradiation (TBI). Deletions of both self-reactive and allo-reactive T cells in the chimera state are mechanisms of tolerance to the donor antigen.

Individual chimerism establishment needs conditioning therapy prior to transplantation in order to prevent rejection of DBMCs and engraftment. Regarding conditioning therapy, full chimerism or mixed chimerism can be used in organ transplantation. However, full chimerism is associated with a higher risk of graft versus host disease (GVHD) and somewhat reduced immunocompetence than mixed chimerism [11].

**FULL CHIMERISM**

Full (macro) chimerism is usually produced after BMT when a related cell type and donor cells are easily detected by flow cytometry at levels of 2% or 3%–100%. The achievement of full chimerism, requires some type of ablative pre-conditioning including TBI or cytoreductive chemotherapy combined with pharmacologic immunosuppressive therapy [12]. Usually conditioning therapy creates a space for engraftment of the donor hematopoietic cells in the recipient’s bone marrow (BM). Therefore, not only hematopoietic stem cells (HSCs) give rise to multi- lineage chimerism but also reactive T cells are deleted. For the first time, in 1995, Billingham and Medawar, using full chimerism idea, transplanted replicating donor hematopoietic cells into immunologically-incompetent neonates to induce tolerance and explained the concept of immunologic cytoreduction followed by hematopoietic reconstitution [9]. Then, animal models demonstrated that addition of immunosuppressive regimen results in long-lasting survival of allografts without using an ongoing immunosuppressant [13]. In both experiments, tolerance is primarily induced and maintained by central deletion of potential donor-reactive T cells, although peripheral mechanisms are also likely to contribute to the process [14].

**MIXED CHIMERISM**

Mixed (micro) chimerism is referred to as “a state where hematopoietic populations of both the recipient and the donor coexist.” Mixed, but not full chimeras, contain a lifelong source of host-type antigen-presenting cells (APC) that can most effectively present antigens to T cells in the recipient thymus [15] and maintains robust tolerance through intrathymic clonal deletion of donor reactive cells, the so-called “central deletion” [16]. In this model, donor cells can only be detected by molecular techniques such as polymerase chain reaction (PCR) and flow-PCR. Some studies have discussed that BM-derived recipient progenitor cells can be incorporated into the transplant after organ transplantation [17,18]. They can adopt various morphological and functional phenotypes, including renal tubular epithelial cells, endothelial cells, interstitial myofibroblasts, hepatocytes, bile duct epithelial cells, cardiomyocytes, pneumocytes, and bronchial epithelial cells [19]. Although trans-differentiation of recipient-derived BMCs into allograft-specific cells or fusion of pluripotent stem cells with respective organ cells are two proposed mechanisms which lead to mixed chimerism [20], the mechanism behind the intriguing phenomenon of non-leukocyte intra-organ mixed chimerism is still not entirely clear.

To achieve successful mixed chimerism and tolerance, certain barriers must be overcome. The most important barrier is mature T cells that have anti-donor reactivity in the periphery and thymus which must be eliminated or inactivated by conditioning the initial host which is accomplished by depleting or using co-stimulatory blockers [21]. Repeated anti-T cell antibody injection [22], and co-stimulatory blockers [23], can eliminate this residual thymic allo-reactivity. Another barrier is memory T cells derived from the response to pathogens which are resistant to T cell depleting antibodies and co-stimulatory blockade may have allogeneic cross-reactivity [24].

Natural killer (NK) cells also possess a barrier to allogeneic BM engraftment by resisting engraftment of pluripotent HSCs, [25,26]. Increased marrow doses cause to overcome this resistance [27], however *in vivo *studies have shown clearly that NK cells develop mutual tolerance in marrow cells [28]. Finally, the last barrier is natural antibodies that can easily be tolerized by the induction of mixed chimerism [29].

**MEASUREMENT OF CHIMERISM**

In the past, chimerism could be measured by numerous methods including cytogenetic (routine banding or fluorescence *in situ* hybridization [FISH] for the Y chromosome in sex-mismatched transplants), red cell phenotyping (in cases of ABO or Rh incompatibility) and restriction fragment length polymorphism (RFLPs) [30]. All of these methods have some limitations. Currently, the most frequently used methods for the measurement of chimerism are X and Y chromosome FISH for gender-mismatched transplants and DNA-based methods for the remaining allogeneic transplants. Southern blot hybridization to detect the RFLPs and variable number of tandem repeat (VNTR) for engraftment monitoring [31]. Since PCR-based techniques require less DNA and can generate results faster, they allow for earlier evaluation of chimerism after the transplant [31]. A recent method is real-time quantitative PCR (qPCR) which is more sensitive (detection of 1/10,000 cells) and can be done within a few hours [32]. However, at higher levels of mixed chimerism, the qPCR is less accurate than the measurement of fluorescent VNTR polymorphic fragments [31]. The PCR-flow technique is another method to detect chimerism that combines the advantage of PCR amplification power by using fluorescent labeled primers to identify single-copy HLA class II DR genes of either donor or recipient origin, together with the ability to bind multiple fluorochrome-labeled (multicolor) CD epitope-specific monoclonal antibodies on intact fixed permeabilized cells [33].

**CLINICAL TRIALS IN INDUCTION OF MIXED CHIMERISM**

In the clinical setting, proof-of-principle for successful tolerance induction or donor-specific hypo-responsiveness by BMC from a cadaver or live related donors has been provided by several reports of sequential allogeneic DBMC which were later followed by a solid organ allograft from the same donor, for a new indication [34]. The final goal of all transplantations is to accomplish a state of permanent tolerance to the allograft in the absence of long-term immunosuppressive therapy. Recently, association between micro-chimerism, tolerance and donor specific hyporesponsiveness has not uniformly substantiated and is controversial. Indeed, in some reports, allograft rejection occurred in the presence of micro-chimerism whereas in other studies, it occurred in the absence of micro-chimerism.

Historically, there have been a small number of patients who have received BMT for hematologic malignancies that later received kidney transplants (KT) from the same donors, without requiring long-term immunosuppression [35,36]. With this knowledge, Monaco and Wood, for the first time demonstrated experimentally in mice that the addition of DBMC to an immunosuppressive regimen, including antilymphocyte serum (ALS), resulted in long-lasting survival of skin allografts without the use of ongoing immunosuppression [37]. Thereafter, several pilot studies on living related donor (LRD) kidney transplantation [38] and cadaver kidney transplantation [39] were performed. In all mentioned models, the recipient’s marrow and immune system were ablated with radiation and/or chemotherapy and replaced entirely with DBMC. However, recipients were at high risk for developing complications due to toxicity of the ablation therapy, and GVHD—both potentially lethal. Recently, with mild ablation protocols to form micro- or mixed-chimerism, it has been suggested that DBMC infusion was clinically safe. Thus, there did not appear to be a need to substantially deviate from an established immunosuppressive protocol such that a perceptible increase in immune reactivity against the donor, while using this immunosuppressive regimen, was not observed [40].

Since then, and nurtured by the micro-chimerism theory of Starzl, *et al* [7], many trials have been performed in attempts to induce donor-specific hyporesponsiveness via donor BM infusion in the kidneys, liver, heart, lungs and pancreatic transplantations (Table 1). In general, good graft survival has been achieved, perhaps with some reduction in chronic rejection, but the clear-cut benefits of additional BM or peripheral stem cell infusion have not been demonstrated.

**Table1:** Outcome of Chimerism induction using different methods

**Protocol**	**Induction**	**Maintenance**	**Patient**	**Follow up**	**Engraftment**	**GVHD**	**Outcome**	**Ref**
DBMI 5×10^8^ cell/kg Central venous 1-2 dose 5-14 days post-operative	OKT3 Tacrolimus AZA or MMF steroids		n = 57 SPK	5 years	Not reported	No GVHD	No difference Chimerism in BM and peripheral blood	[76-78]
CAD 5×10^8^ cell/kg 1 or 2 injection 2 weeks post-operative Minimum of 1 DR matching	OKT3	tacrolimus, AZA or mycophenolate mofetil (MMF), and steroids	Kidney	6 years	Not reported	Not reported	No difference	[43]
CAD DBMC 7.01±1.9 ×10^8 ^ cell/kg Day 4 and 11 or Day 4 post-operative	OKT3 Tacrolimus MMF	Methyl prednisolone	n = 63 kidney	2.5–5.5 years	Not reported	Not reported	Modulatory effect on chronic rejection and allograft survival.	[79]
LRD 1.8±1.9 ×10^8^ cell/kg 4 days post-operative	OKT3 or Daclizumab	Tacrolimus Mycophenolate mofetil Methyl prednisolone	n = 47	19–61 months	Not reported	Not reported	Decreasing immunosuppressive dosage	[80]
CD34 mobilized PSCs HLA mismatched	ATG TLI	Cyclosporine Prednisolone	n = 4 kidney	Up to 3 years	3/4 All lost by 3 months post-operative	None	2/4 weaned of all immunosupression No tolerance achieved	[81]
Intrathymic High dose PBSCs Portal and systemic 5 HLA matched 28 HLA mismatched	Cyclosphamide ATG	Cyclosporine Prednisolone	n = 33 kidney	Up to 210 days	Not reported	No reported	4 patients weaned of from all immunosupression and rejection free	[82,48]
DBM HLA identical	Cyclophosamide ATG TLI	Cyclosporine DLI for patients with mixed chimerism	n = 6 kidney	Up to 9 years	6/6	2/6 GVHD	6/6 tolerant 1/6 cellular rejection	[83]
DBM HLA mismatched	Cyclophosamid Rituximab Siplizumab Prednisolon TI	Cyclosphorin	n = 5	Up to 5 years	5/5 with initial micro-chimerism All lost by 3 weeks	None	4/5 tolerant Antibody rejection	[84]
CD34 mobilized PSCs HLA identical	ATG TLI	Cyclosporine MMF Prednisolon	n = 3 kidney	Up to 2 years	2/3	None	1/3 weaned of all immunosupression and tolerant 1/3 weaned with subsequent rejection 1/3 not weaned because of recurrent FSGS	[85]

**DONOR BM INFUSION IN KIDNEY TRANSPLANTS**

More than 60 years ago, it was found that mixed hematopoietic chimerism, when established in the fetus or neonate, leads to transplantation tolerance. In the mid-1970s, Monaco, *et al*, the pioneer of DBMI in kidney transplantation (KT), used anti-lymphocyte globulin (ALG) for a two-week induction therapy in renal transplantation followed by conventional immunosuppression with prednisone, azathioprine (AZA) and donor BM cells infusion from the LRD-iliac crest 21 days post-KT. They subsequently detected reduced *in vitro *anti-donor activity [41]. Barber, *et al*, who analyzed 57 cadaveric renal allograft recipients who underwent immunosuppression with ALG, cyclosporine, azathioprine and prednisone and received cryopreserved DBMI 21 days post-KT, were compared to a control group of 54 kidney recipients who had identical immunosuppression, but no BM infusion. The differences between the control and test groups were striking: three graft losses and one chronic rejection in the BM group when compared to 13 graft losses and five chronic rejections in the control group. Numerous patients in the BM group could be tapered off prednisone; however, operational tolerance with the ability to discontinue all immunosuppression was not demonstrated [42]. Chronic rejection was significantly less in the DBM group than in a concurrent, non-randomized control group in a six-year follow-up of patients who received DBMI and KT [43]. In this study, OKT3 (anti-T cell antibody) and maintenance therapy with tacrolimus, AZA or mycophenolate mofetil (MMF), and steroids were used. The presence of donor CD34+ in the DBM group has been shown by flow-PCR in the iliac crest BM aspirate recipients contained an average of 13 times more donor CD34+ cells than in the peripheral blood (PB), which supporting the idea of engraftment [44,45]. 

In another clinical trial, Trivedi developed a preoperative, non-myeloablative, mega-dose, unfractionated HSC infusion protocol to create tolerance in cadaver renal allograft recipients who had no GVHD or hepatic dysfunction and were almost AR-free. These patients had stable graft function and a very low incidence of CMV disease, with minimal immunosuppression [46]. Experiments have proved that donor-specific hyporesponsiveness could be achieved by intrathymic inoculation of donor alloantigens [47]. Thus, Trividi and colleagues transplanted donor HSC intrathymic in 66 patients who were scheduled for living-donor KT [48]. Donor-specific transfusions and high-dose HCT were applied to both peripheral and portal circulation as well as an intra-osseous injection. A reduced intensity conditioning regimen including cyclophosphamide, T cell depletion with ATG and localized low dose irradiation (abdominal and inguinal lymph nodes, thoracolumbar vertebrae and a portion of the pelvis) were used. KT was performed after a documented consecutive negative cross-match. In this clinical trial, four patients were completely weaned off all immunosuppression with no rejection for up to 210 days. However, no details on chimerism and GVHD or any functional immunologic assays were reported. 

In a study by Leung, eight patients underwent KT with immediate graft function. However, only five of these subsequently received successful HSC transplantation with satisfactory trilineage engraftment [49]. The most common complications during stem cell transfusion in solid organ transplantation were neutropenic fever or bacteremia, and gastrointestinal disturbances.

In another pilot study of five highly selected renal transplantation patients who underwent mixed chimerism induction with a non-myeloablative conditioning regimen, four were functionally tolerant 2 to 5.3 years following complete withdrawal of immunosuppressive drugs [50].

**CONTEMPLATIONS RELATIVE TO INDUCTION OF TOLERANCE BY HEMATOPOIETIC CHIMERISM**

Since the clinical development of chimerism can be a possible route towards tolerance in solid organ transplantation, it is useful to consider suggestions regarding how it might be achieved.

Source of donor cells: This includes unmodified BM mononuclear cells and the progenitors from PB, with or without cytokine stimulation (*e.g.*, nupogen). For induction of mixed chimerism, the percentage of CD34+ is likely to be important. Additionally, the presence of dendritic cells, immunoregulatory cells or other stromal cells may facilitate chimerism induction.

Timing: When should BMSCs be administered—prior to, during, or post-organ transplantation? Infusion of BM three weeks post-transplantation has not had any significant clinical effects, so in the majority of protocols, it is administered sooner. It has been suggested that for induction of mixed chimerism, the time interval between stem cell and organ transplantation should be approximately four to six weeks post-operative to allow for recovery of hematopoiesis before transplant surgery. Infusion of HSCs during the organ transplant or pre-operation were also reported in some studies.

Number of infusions: Currently, there is no protocol superior to others.

Dose of cells: Generally 5×10^8^ nucleated cells/kg or 5×10^6^ CD34+ cells/kg from the recipients is suggested, with resultant micro-chimerism, however mega-doses would be better.

Ablative therapy: Which ablative therapy should be used? Candidate antibodies might include OKT3, ATG, anti-CD4, anti-CDS, or antibodies to co-stimulatory molecules. The use of antibodies decreases the incidence of GVHD. Another approach is “prope tolerance” as described by Calne with preconditioning using the monoclonal antibody Campath-1H. Campath has been established as a powerful regimen that depletes T and B lymphocytes, and monocytes, but not BMSCs.

Route of administration: Bone marrow stem cells should be given through peripheral blood via a central line, peripheral access or intra-portally. Some data have suggested that the portal route increases the likelihood of tolerance. In addition, intra-BM injection or isolated limb perfusion is more efficient than the intravenous route for achieving engraftment of donor HSCs [51].

Percentage of HLA matching: To achieve chimerism or tolerance, it is necessary to have at least one matched DR antigen.

Useful *in vitro* tolerance assay: Despite the availability of mixed lymphocyte reaction (MLR), cell-mediated lymphocytotoxicity (CML) assays, and precursor CTL assay which has a higher level of predictability, there is no single or collection of *in vitro* tests that correlate with the level of *in vivo* tolerance. Moreover, there is a sense that the presence of chimerism/micro-chimerism may be a marker of tolerance [52]. However, this concept has been questioned as perhaps an “epiphenomenon.” The use of quantitative levels of micro-chimerism as a measure of donor unresponsiveness and a possible guide for discontinuing immunosuppression has been described as a kind of “madness” [7], that may be necessary to effect a change in conventional thinking.

**MESENCHYMAL STEM CELLS**

MSCs, described initially by Friedenstein in 1970, are multipotent cells which can be isolated from BM by their ability to adhere to plastic. MSCs can be differentiated into various mesodermal cell lineages and obtained from a variety of tissues such as BM, PB, umbilical cord blood, placenta, and many others. MSCs, as multipotent cells, are defined according to phenotype, and growth and differentiation characteristics. MSCs differentiate into multiple different cell lineages producing important growth factors and cytokines that may facilitate repair of damaged tissues. However, MSCs demonstrate unique immunomodulatory properties and have emerged as promising candidates for cell-based immunotherapy by promoting tolerance of solid allografts. MSCs modulate the immune response in various ways and *in vitro* experiments have demonstrated their non-immunogenic and immunosuppressive characteristics. Although other stromal cell-like fibroblasts show similar effects; with respect to suppressing T cell proliferation in a clinically significant way, MSCs compete with other cell populations. In addition to the immunomodulation effects of MSCs with respect to expanded, production, and storage in the large quantities needed for therapeutic trials, they have been applied successfully in experimental solid organ transplantation and clinical studies.

**IMMUNOMODULATORY PROPERTIES OF MSCS**

The *in vitro* immunomodulatory properties of MSCs were initially demonstrated with T cell response inhibition, and initially described in a skin transplantation model after which they were applied to attenuate GVHD. Numerous studies have demonstrated that MSCs inhibit the T cell response in MLR [53-57]. MSCs interact with T cells and activate regulatory T cells (CD4/CD25 double-positive) [55], which can inhibit the immune response both *in vitro* and *in vivo* to prolong the survival of skin allografts [54], and attenuate GVHD after BMT [58-62].

MSCs-secreted soluble factors such as prostaglandin E2 (PGE2) can inhibit T cell proliferation *in vitro*. Aggarwal and Pittenger have shown that addition of the PGE2 synthesis inhibitor, indomethacin, partially restored T cell proliferation in their model. MSCs can produce nitric oxide (NO) to suppress T cell proliferation [57]. Suppression of NO production in MSCs reduces the inhibition capacity of T cell proliferation. Indoleamine 2,3-dioxygenase (IDO), a cytoplasmic enzyme that converts the essential amino acid tryptophan to kynurenine, is produced by MSCs upon stimulation with interferon (IFN)-β and inhibits T cell proliferation in MLR [63]. IDO play a key role in the establishment and maintenance of peripheral tolerance. It has a major T cell inhibitory activity in an APC/T cell interaction and has been identified as the controlling mechanism for fetal acceptance during pregnancy [64]. Direct inhibition of T cell proliferation by IDO might prolong allograft survival *in vivo* [65,66]. However, Gieseke, *et al*, have shown that MSCs with a mutation in the IFN-β receptor, cannot express IDO efficiently and inhibit the PB monocyte proliferation [67].

Taking into account all available data, it can be shown that there is a complex interaction between MSCs and the immune system which results in T cell proliferation. This property makes them a good candidate for immune modulation in allograft transplantation.

**MSCS IN ORGAN TRANSPLANTATION**

Lazarus, *et al*, in 1995, initially reported intravenous infusion of human BM-derived MSCs for patients who underwent BMT [68]. Examination of their BM showed no adverse reactions with infusion of *ex vivo* expanded MSCs. The same group subsequently conducted a phase I-II clinical trial to determine BM-derived MSCs on hematopoietic rescue in patients with breast cancer after high dose chemotherapy [69]. The results were the same as the first study in that no adverse effects were observed in the case group. However, the study could not prove the effects of MSCs on hematopoietic recovery after transplantation. Five years later, based on studies that have shown the effect of MSCs through multiple mechanisms in which they have the potential to effect immunological, inflammatory, vascular, and regenerative pathways, it has been suggested that their immunomodulatory properties could be used in solid organ transplantation for prevention and/or treatment of organ rejection [70].

Although there are no published clinical data on the use of MSCs in organ transplantation, animal studies indicate that MSCs might interfere with ischemic, inflammatory, and immunological mechanisms after organ transplantation and their beneficial effects may arise through multiple mechanisms. Morigi, *et al*, have shown that intravenously- or locally-injected MSCs in a cisplatin-induced renal injury model improved organ function through cell engraftment in damaged kidneys and differentiated into tubular epithelial cells [71]. However, other investigators have shown that this improvement arises solely from paracrine effects [72,73]. There are no reports on the effect of MSCs in an animal KT model, but theoretically the immunomodulatory capabilities of MSCs could prevent and/or treat AR after KT.

AR after OLT can almost be controlled with large doses of immunosuppressants that have severe toxic as well as other side effects. Therefore, the use of MSCs could be a safe and effective method for preventing and treating rejection. Wang, *et al*, have investigated the immunoregulatory effects of rat MSCs in a model of allogeneic liver transplantation [74]. Infusions of BM-derived MSCs from three different sources (recipient, donor, or third-party rats) induced generation of CD4+ CD25+ Foxp3+ Tregs and markedly prolonged graft survival when compared with control animals. The same results were seen in a study by Wan, *et al*, where adipose-derived MSCs in a rat model of allogeneic liver transplantation were used [75]. The researchers showed that adipose-derived MSCs clearly inhibited recipient-derived T lymphocyte proliferation in an MLR and significantly alleviated AR following OLT.

Much of our knowledge of MSCs is based on *in vitro* experiments. We need to conduct more research to understand the mechanisms through which MSCs mediate their apparent beneficial effects in immunomodulation for organ transplantation. Most of known characteristics of MSCs are based on *in vitro* culture and may not have any *in vivo* counterparts. Doing larger clinical trials could validate their applicability as therapeutic modalities in transplantation.

## CONCLUSIONS

Today, we still know little about the exact tolerogenic effects of mixed chimerism or functional role(s) of MSCs *in vivo* in health and disease conditions. However, these questions have not deterred clinicians from testing these modalities in several clinical applications. Recently, Kawai, *et al*, demonstrated that induction of mixed chimerism following combined BM and kidney transplants from HLA single-haplotype mismatched donors could discontinue all immunosuppressive therapy, without significantly affecting transplant function [50]. As well as mixed chimerism, using of MSCs to accelerate hematopoietic engraftment after transplantation has now expanded to testing their potential to differentiate into and/or participate in organ transplantation. Although results obtained so far indicate that significant hurdles still need to be overcome before organ transplant recipients can be weaned off drugs safely and routinely, these new directions may ultimately capture the elusive state of alloimmune tolerance.

## References

[1] Joseph J, Baines L, Morris M (2003). Quality of life after kidney and pancreas transplantation. Am J Kidney Dis.

[2] Sayegh M, Carpenter C (2004). Transplantation 50 years later--progress, challenges, and promises. N Engl J Med.

[3] Bloom R, Doyle A (2006). Kidney disease after heart and lung transplantation. Am J Transplant.

[4] Kasiske B, Anjum S, Shah R (2004). Hypertension after kidney transplantation. Am J Kidney Dis.

[5] Tantravahi J, Womer K, Kaplan B (2007). Why hasn’t eliminating acute rejection improved graft survival?. Annu Rev Med.

[6] Reinsmoen N, Hertz M, Kubo S (1993). Immune factors correlating with improved long-term graft outcome in kidney, lung, and heart transplants. Transplant Proc.

[7] Starzl T, Demetris A, Murase N (1996). The lost chord: microchimerism and allograft survival. Immunol Today.

[8] Lion T (2007). Detection of impending graft rejection and relapse by lineage-specific chimerism analysis. Methods Mol Med.

[9] BILLINGHAM R, BRENT L, MEDAWAR P (1953). Actively acquired tolerance of foreign cells. Nature.

[10] Starzl T, Zinkernagel R (2001). Transplantation tolerance from a historical perspective. Nat Rev Immunol.

[11] Sykes M, Sachs D (1988). Mixed allogeneic chimerism as an approach to transplantation tolerance. Immunol Today.

[12] Okayama J, Ko S, Kanehiro H (2004). Bone marrow chimerism and tolerance induced by single-dose cyclophosphamide. J Surg Res.

[13] Horton PJ, Hawthorne WJ, Walters S (2008). Tolerance induction in a large-animal model using total lymphoid irradiation and intrathymic bone marrow. Transplantation.

[14] Ciancio G, Burke GW, Moon J (2004). Donor bone marrow infusion in deceased and living donor renal transplantation. Yonsei Med J.

[15] Sykes M (2007). Immune tolerance: mechanisms and application in clinical transplantation. J Intern Med.

[16] Lan T, Chen J, Xia J (2010). Inhibition of alloantigen-primed memory CD4+ and CD8+ T cells by hematopoietic chimerism in mice. Scand J Immunol.

[17] Mengel M, Jonigk D, Wilkens L (2004). Chimerism of metanephric adenoma but not of carcinoma in kidney transplants. Am J Pathol.

[18] Xu Q, Lee J, Keller M (2009). Analysis of indirect pathway CD4+ T cells in a patient with metastable tolerance to a kidney allograft: possible relevance to superior graft survival of HLA class II closely matched renal allografts. Transpl Immunol.

[19] Kørbling M, Estrov Z (2003). Adult stem cells for tissue repair - a new therapeutic concept?. N Engl J Med.

[20] Alvarez-Dolado M, Pardal R, Garcia-Verdugo J (2003). Fusion of bone-marrow-derived cells with Purkinje neurons, cardiomyocytes and hepatocytes. Nature.

[21] Setoguchi K, Kishimoto H, Kobayashi S (2010). Potential role of host effector memory CD8(+) T cells in marrow rejection after mixed chimerism induction in cynomolgus monkeys. Transpl Immunol.

[22] Nikolic B, Khan A, Sykes M (2001). Induction of tolerance by mixed chimerism with nonmyeloblative host conditioning: the importance of overcoming intrathymic alloresistance. Biol Blood Marrow Transplant.

[23] Verbinnen B, Van Gool S, Ceuppens J (2010). Blocking costimulatory pathways: prospects for inducing transplantation tolerance. Immunotherapy.

[24] Xie B, Chen J, Xia J (2009). Combined costimulation blockade inhibits accelerated rejection mediated by alloantigen-primed memory T cells in mice. Immunol Invest.

[25] Nierlich P, Klaus C, Bigenzahn S The role of natural killer T cells in costimulation blockade-based mixed chimerism. Transpl Int.

[26] van der Touw W, Bromberg J (2010). Natural killer cells and the immune response in solid organ transplantation. Am J Transplant.

[27] Lee JM, Lee CJ, Hu CY (1996). Chimerism in survivors following allograft renal transplantation. Transplant Proc.

[28] Zhao Y, Ohdan H, Manilay J (2003). NK cell tolerance in mixed allogeneic chimeras. J Immunol.

[29] Ohdan H, Yang Y, Shimizu A (1999). Mixed chimerism induced without lethal conditioning prevents T cell- and anti-Gal alpha 1,3Gal-mediated graft rejection. J Clin Invest.

[30] Bader P, Niethammer D, Willasch A (2005). How and when should we monitor chimerism after allogeneic stem cell transplantation?. Bone Marrow Transplant.

[31] Liesveld J, Rothberg P (2008). Mixed chimerism in SCT: conflict or peaceful coexistence?. Bone Marrow Transplant.

[32] Kristt D, Gesundheit B, Stein J (2010). Quantitative monitoring of multi-donor chimerism: a systematic, validated framework for routine analysis. Bone Marrow Transplant.

[33] Garcia-Morales R, Esquenazi V, Carreno M (1997). PCR-flow analysis used to detect the levels of chimerism in peripheral blood of bone-marrow infused organ allograft recipients at the time of rejection episodes. Transplant Proc.

[34] Chen J, Kuo M, Ou L Characterization of tolerance induction through prenatal marrow transplantation: the requirement for a threshold level of chimerism to establish rather than maintain postnatal skin tolerance. Cell Transplant.

[35] Jacobsen N, Taaning E, Ladefoged J (1994). Tolerance to an HLA-B,DR disparate kidney allograft after bone-marrow transplantation from same donor. Lancet.

[36] Spitzer TR, Delmonico F, Tolkoff-Rubin N (1999). Combined histocompatibility leukocyte antigen-matched donor bone marrow and renal transplantation for multiple myeloma with end stage renal disease: the induction of allograft tolerance through mixed lymphohematopoietic chimerism. Transplantation.

[37] Monaco A, Wood M (1970). Studies on heterologous antilymphocyte serum in mice. VII. Optimal cellular antigen for induction of immunologic tolerance with antilymphocyte serum. Transplant Proc.

[38] De Fazio S, Hartner W, Monaco A (1985). Mouse skin graft prolongation with donor strain bone marrow and antilymphocyte serum. Effect of donor age. Transplantation.

[39] Barber W, Mankin J, Laskow D (1991). Long-term results of a controlled prospective study with transfusion of donor-specific bone marrow in 57 cadaveric renal allograft recipients. Transplantation.

[40] Hawksworth J, Leeser D, Jindal R (2009). New directions for induction immunosuppression strategy in solid organ transplantation. Am J Surg.

[41] Monaco A, Clark AW, Wood M (1976). Possible active enhancement of a human cadaver renal allograft with antilymphocyte serum (ALS) and donor bone marrow: case report of an initial attempt. Surgery.

[42] Barber WH, Hudson SL, Deierhoi MH (1990). Donor antigen-specific immunosuppression in cadaveric and living-related donor kidney allograft recipients. Clin Transpl.

[43] Garcia-Morales R, Carreno M, Mathew J (1997). The effects of chimeric cells following donor bone marrow infusions as detected by PCR-flow assays in kidney transplant recipients. J Clin Invest.

[44] Garcia-Morales R, Esquenazi V, Zucker K (1997). Assessment of the effects of cadaveric donor bone marrow on chimerism in kidney transplant recipients by the polymerase chain reaction-flow technique. Transplant Proc.

[45] Mathew JM, Garcia-Morales R, Fuller L (2000). Donor bone marrow-derived chimeric cells present in renal transplant recipients infused with donor marrow. I. Potent regulators of recipient antidonor immune responses. Transplantation.

[46] Trivedi H, Shah V, Shah P (2001). Megadose approach to DBMC infusion-induced allograft hyporesponsiveness in living-related renal allograft recipients. Transplant Proc.

[47] Remuzzi G, Perico N (1995). Immunotolerance: from new knowledge of mechanisms of self-tolerance to future perspectives for induction of renal transplant tolerance. Exp Nephrol.

[48] Trivedi HL, Vanikar AV, Modi PR (2005). Allogeneic hematopoietic stem-cell transplantation, mixed chimerism, and tolerance in living related donor renal allograft recipients. Transplant Proc.

[49] Leung N, Dispenzieri A, Fervenza F (2005). Renal response after high-dose melphalan and stem cell transplantation is a favorable marker in patients with primary systemic amyloidosis. Am J Kidney Dis.

[50] Kawai T, Benedict CA (2010). Induction of tolerance in clinical kidney transplantation. Clin Transplant.

[51] Askenasy N, Zorina T, Farkas D (2002). Transplanted hematopoietic cells seed in clusters in recipient bone marrow in vivo. Stem Cells.

[52] Mathew J, Miller J (2001). Immunoregulatory role of chimerism in clinical organ transplantation. Bone Marrow Transplant.

[53] Kamel S, Wood G (1990). Immunoregulatory activity of cells from lymph nodes draining the uterus of allopregnant mice. J Reprod Immunol.

[54] Bartholomew A, Sturgeon C, Siatskas M (2002). Mesenchymal stem cells suppress lymphocyte proliferation in vitro and prolong skin graft survival in vivo. Exp Hematol.

[55] Krampera M, Glennie S, Dyson J (2003). Bone marrow mesenchymal stem cells inhibit the response of naive and memory antigen-specific T cells to their cognate peptide. Blood.

[56] Le Blanc K, Tammik L, Sundberg B (2003). Mesenchymal stem cells inhibit and stimulate mixed lymphocyte cultures and mitogenic responses independently of the major histocompatibility complex. Scand J Immunol.

[57] Aggarwal S, Pittenger M (2005 ). Human mesenchymal stem cells modulate allogeneic immune cell responses. Blood.

[58] Le Blanc K, Ringdén O (2005). Immunobiology of human mesenchymal stem cells and future use in hematopoietic stem cell transplantation. Biol Blood Marrow Transplant.

[59] Le Blanc K, Ringdén O (2006). Mesenchymal stem cells: properties and role in clinical bone marrow transplantation. Curr Opin Immunol.

[60] Le Blanc K, Ringdén O, Review N (2007). Immunomodulation by mesenchymal stem cells and clinical experience. J Intern Med.

[61] Tian Y, Deng Y, Huang Y (2008). Bone marrow-derived mesenchymal stem cells decrease acute graft-versus-host disease after allogeneic hematopoietic stem cells transplantation. Immunol Invest.

[62] von Bonin M, Stölzel F, Goedecke A (2009). Treatment of refractory acute GVHD with third-party MSC expanded in platelet lysate-containing medium. Bone Marrow Transplant.

[63] Opitz C, Litzenburger U, Lutz C (2009). Toll-like receptor engagement enhances the immunosuppressive properties of human bone marrow-derived mesenchymal stem cells by inducing indoleamine-2,3-dioxygenase-1 via interferon-beta and protein kinase R. Stem Cells.

[64] Barry F, Murphy J, English K (2005). Immunogenicity of adult mesenchymal stem cells: lessons from the fetal allograft. Stem Cells Dev.

[65] Quan J, Tan P, MacDonald A (2008). Manipulation of indoleamine 2,3-dioxygenase (IDO) for clinical transplantation: promises and challenges. Expert Opin Biol Ther.

[66] Jia L, Tian P, Ding C (2009). Immunoregulatory effects of indoleamine 2, 3-dioxygenase in transplantation. Transpl Immunol.

[67] Gieseke F, Schütt B, Viebahn S (2007). Human multipotent mesenchymal stromal cells inhibit proliferation of PBMCs independently of IFNgammaR1 signaling and IDO expression. Blood.

[68] Lazarus H, Haynesworth S, Gerson S (1995). Ex vivo expansion and subsequent infusion of human bone marrow-derived stromal progenitor cells (mesenchymal progenitor cells): implications for therapeutic use. Bone Marrow Transplant.

[69] Koç O, Gerson S, Cooper B (2000). Rapid hematopoietic recovery after coinfusion of autologous-blood stem cells and culture-expanded marrow mesenchymal stem cells in advanced breast cancer patients receiving high-dose chemotherapy. J Clin Oncol.

[70] Devine S, Hoffman R (2000). Role of mesenchymal stem cells in hematopoietic stem cell transplantation. Curr Opin Hematol.

[71] Morigi M, Imberti B, Zoja C (2004). Mesenchymal stem cells are renotropic, helping to repair the kidney and improve function in acute renal failure. J Am Soc Nephrol.

[72] Tögel F, Hu Z, Weiss K (2005). Administered mesenchymal stem cells protect against ischemic acute renal failure through differentiation-independent mechanisms. Am J Physiol Renal Physiol.

[73] Kunter U, Rong S, Djuric Z (2006). Transplanted mesenchymal stem cells accelerate glomerular healing in experimental glomerulonephritis. J Am Soc Nephrol.

[74] Wang D, Zhang H, Liang J (2010). Effect of allogeneic bone marrow-derived mesenchymal stem cells transplantation in a polyI:C-induced primary biliary cirrhosis mouse model. Clin Exp Med.

[75] Wang Y, Zhang A, Ye Z (2009). Bone marrow-derived mesenchymal stem cells inhibit acute rejection of rat liver allografts in association with regulatory T-cell expansion. Transplant Proc.

[76] Burke G, Ricordi C, Karatzas T (1995). Donor bone marrow infusion in simultaneous pancreas/kidney transplant recipients: a preliminary study. Transplant Proc.

[77] Burke G, Ricordi C, Karatzas T (1997). Donor bone marrow infusion in simultaneous pancreas/kidney transplantation with OKT3 induction: evidence for augmentation of chimerism. Transplant Proc.

[78] Burke G, Ciancio G, Garcia-Morales R (1998). Evidence for microchimerism in peripheral blood, bone marrow, and skin following donor bone marrow/kidney-pancreas transplantation at 3 years. Transplant Proc.

[79] Ciancio G, Miller J, Garcia-Morales RO (2001). Six-year clinical effect of donor bone marrow infusions in renal transplant patients. Transplantation.

[80] Ciancio G, Burke GW, Garcia-Morales R (2002). Effect of living-related donor bone marrow infusion on chimerism and in vitro immunoregulatory activity in kidney transplant recipients. Transplantation.

[81] Millan MT, Shizuru JA, Hoffmann P (2002). Mixed chimerism and immunosuppressive drug withdrawal after HLA-mismatched kidney and hematopoietic progenitor transplantation. Transplantation.

[82] Trivedi H, Vanikar A, Vakil J (2004). A strategy to achieve donor-specific hyporesponsiveness in cadaver renal allograft recipients by donor haematopoietic stem cell transplantation into the thymus and periphery. Nephrol Dial Transplant.

[83] Fudaba Y, Spitzer TR, Shaffer J (2006). Myeloma responses and tolerance following combined kidney and nonmyeloablative marrow transplantation: in vivo and in vitro analyses. Am J Transplant.

[84] Kawai T, Cosimi AB, Spitzer TR (2008). HLA-mismatched renal transplantation without maintenance immunosuppression. N Engl J Med.

[85] Scandling JD, Busque S, Dejbakhsh-Jones S (2008). Tolerance and chimerism after renal and hematopoietic-cell transplantation. N Engl J Med.

